# Visceral Fat Accumulation, Insulin Resistance, and Elevated Depressive Symptoms in Middle-Aged Japanese Men

**DOI:** 10.1371/journal.pone.0149436

**Published:** 2016-02-18

**Authors:** Shuichiro Yamamoto, Yumi Matsushita, Toru Nakagawa, Toru Honda, Takeshi Hayashi, Mitsuhiko Noda, Tetsuya Mizoue

**Affiliations:** 1 Occupational Health Section, Hitachi, Ltd., Hitachi Health Care Center, Hitachi city, Ibaraki, Japan; 2 Department of Clinical Research, Center for Clinical Sciences, National Center for Global Health and Medicine, Shinjuku-ku, Tokyo, Japan; 3 Department of Endocrinology and Diabetes, Saitama Medical University, Iruma-gun, Saitama, Japan; 4 Department of Epidemiology and Prevention, Center for Clinical Sciences, National Center for Global Health and Medicine, Shinjuku-ku, Tokyo, Japan; National Institute for Viral Disease Control and Prevention, CDC, China, CHINA

## Abstract

**Objective:**

To investigate visceral fat accumulation and markers of insulin resistance in relation to elevated depressive symptoms (EDS).

**Methods:**

Participants were 4,333 male employees (mean age, 49.3 years) who underwent abdominal computed tomography scanning, measured fasting insulin, and did not self-report diabetes and mental disorders under treatment and history of cancer, myocardial infarction, and stroke. Multivariable logistic regression was used to assess the association of EDS with abdominal fat deposition and markers of insulin resistance.

**Results:**

Visceral fat area (VFA) and fasting insulin were significantly, positively associated with EDS. Multivariable-adjusted odds ratios (95% confidence interval) of high VFA for the lowest through highest quartile of depression score were 1 (reference), 1.18 (0.97–1.42), 1.25 (1.02–1.54), 1.23 (1.01–1.51), respectively, and corresponding figures for high fasting insulin were 1 (reference), 0.98 (0.80–1.19), 1.12 (0.91–1.38), and 1.29 (1.06–1.57), respectively. Subcutaneous fat area was not associated with EDS.

**Conclusions:**

Results suggest that EDS is related to visceral, but not subcutaneous, fat accumulation and insulin resistance in middle-aged Japanese men.

## Introduction

Numerous studies have reported the association of obesity or diabetes with depression [1–6]. Depression is associated with an increased risk of diabetic complications and mortality among patients with diabetes [7,8]. The relationship between depression and obesity or diabetes may be bidirectional [5,6]. Potential mechanisms underlying these associations include hypothalamic-pituitary-adrenal axis (HPA-axis) dysregulation with hypercortisolemia [9], inflammation [10], influences of lifestyle behaviors [11,12] and side effect of antidepressants [13].

In spite of accumulating epidemiological and experimental evidence linking depression to obesity, it remains unclear which fat deposition is associated with depression. Some studies reported a significant association between abdominal obesity (waist-hip ratio or waist circumference) and depression [14–16], suggesting a role of abdominal adiposity in mood regulation. In small-scale studies with the use of computed tomography (CT) or magnetic resonance tomography, depressed patients had higher visceral fat mass than non-depressed individuals [17–19]. In a few large-scale population-based studies in Western countries that measured abdominal fat deposition with the use of CT [20–22], depressive symptoms were associated with visceral adipose accumulation; of these, one study additionally assessed subcutaneous fat [20] but found no association with depressive symptoms. Given that Japanese men have a larger visceral adipose tissue than Caucasian men in the same levels of waist circumference [23], it would be of interest to examine the association between abdominal adiposity and mood among the Japanese population. As regards glucose metabolism, it remains unclear whether pre-diabetic status including insulin resistance is associated with depression [24]. To address these issues, we investigated cross-sectionally the association of elevated depressive symptoms (EDS) with abdominal fat deposition measured with CT and markers of insulin resistance among Japanese men.

## Methods

### Study participants

The Hitachi Health Study is an ongoing study among employees (including retired employees) and their spouses who underwent a comprehensive health checkup at Hitachi Health Care Center (Hitachi, Japan), as described elsewhere [25,26]. Of 17,606 examinees during 2008 fiscal year (between April 2008 and March 2009), 6,996 underwent an abdominal CT. Of these, 4,971 non-retired employees received health examinations in fasting condition (fasted at least 12 h) and had data on all variables used in the present study. We excluded 251 patients with diabetes under treatment, 87 patients with mental disorders under treatment, 73 with history of cancer, 20 with history of myocardial infarction and 20 with history of cerebrovascular disease. We excluded 187 women, leaving 4,333 men for analysis. The objective and procedure of the study were announced by using explanatory leaflet and poster. Written informed consent was obtained from each participant who agreed to provide his/her data for research. The study protocol was approved by the Ethics Committee of the Hitachi Health Care Center and the Ethics Committee of National Center for Global Health and Medicine, Japan.

### Assessment of exposure (depressive symptoms)

Physical and psychological symptoms were assessed using a questionnaire that was developed for health checkup. From that questionnaire, 13 questions on sleep disturbance, poor appetite, gloomy feeling, diminished interest, fatigue, daily fluctuation, irritation and indecision (Table 1) were selected to assess depression status, according to an advice of a psychiatrist. These questions are similar to those of widely-used depression scales including the Zung Self-rating Depression Scale (SDS) or the Center for Epidemiologic Studies Depression Scale (CES-D). Specifically, 7 questions selected (‘No1’, ‘No 3’, ‘No 5’, ‘No 9’, ‘No 10’, ‘No11’ and ‘No 12’) were similar to those in SDS and 5 (‘No 7’, ‘No 8’, ‘No 9’, ‘No11’and ‘No12’) to those in CES-D. Respondents were asked to choose their answer from among 4 response options (‘1 no’, ‘2 sometimes’, ‘3 often’ and ‘4 always’) for the questions ‘No. 1’ to ‘No. 12’ and among 3 options (‘1 no’, ‘2 yes’ and ‘3 severe’) for the question ‘No. 13’. In order to assign an equal weight to all these questions, the scores of 2 and 3 for the question ‘No. 13’ were converted to 2.5 and 4, respectively. Depression score was then calculated as the sum of the scores across the questions. Pearson correlation coefficient between the score calculated from the selected questions and SDS score was 0.752 [27].

**Table 1:** Questions used for assessing depression status.

1. I have an unpleasant awakening due to feeling tired.
2. I can not work efficiently before lunch time.
3. I feel tired and sluggish.
4. I am very concerned about any changes in my physical condition.
5. I easily get irritated.
6. I get anxious for no particular reasons.
7. I have difficulties feeling close to others and feel isolated.
8. I do not feel refreshed and feel gloomy.
9. I can not think clearly and have trouble making a decision.
10. I feel too lazy and am not interested in the things I used to like to do.
11. I have trouble sleeping.
12. I have no appetite.
13. I have a bad headache or feel severely heavy-headed.

### Assessment of outcomes (abdominal obesity and glucose metabolism)

The visceral fat area (VFA) and subcutaneous fat area (SFA) at the umbilical level was measured using a CT scanner (Radix turbo; Hitachi Medico, Tokyo, Japan) while the examinee was in a supine position and estimated using a PC software (fatPointer; Hitachi Medico, Tokyo, Japan). The imaging conditions were 120 kV and 50 mA, using a 5-mm-thick slice. Glucose was measured using glucose oxidase enzyme electrode method (A&T, Tokyo, Japan). Serum insulin (μU/mL) was determined by an immunoenzymatic method using the AxSYM insulin assay (Abbott Laboratories, Tokyo, Japan). Homeostasis model assessment of insulin resistance (HOMA-IR), an index of insulin resistance, was calculated as fasting glucose (mg/dL) multiplied by fasting insulin (μU/mL) divided by 405. Hemoglobin A1c (Japan Diabetes Society [JDS]) was measured using a high-performance liquid chromatography method (HLC723-G9; Tosoh, Tokyo, Japan) and converted to National Glycohemoglobin Standardization Program equivalent value according to the formula: Hemoglobin A1c (%) = Hemoglobin A1c (JDS) (%) + 0.4% [28]. VFA, SFA, fasting insulin and HOMA-IR were categorized into quartiles and the highest groups were defined as outcomes which were named as follows: ‘high VFA’, ‘high SFA’,’ high fasting insulin’ and ‘high HOMA-IR’. ‘Overweight/obesity’ (BMI ≥25 kg/m^2^) and ‘high fasting glucose’ (fasting plasma glucose ≥110 mg/dl) were also defined as outcomes.

### Assessment of other variables

Health-related lifestyles including smoking, alcohol drinking, as well as current and past history of diseases including diabetes, cancer, myocardial infarction, stroke and mental disorder were ascertained via a questionnaire. As regards physical activity, walking commuting to and from work (min/day) and up to three leisure-time physical activities (frequency and duration of time/day) were asked. MET value (MET-min/week) was calculated by multiplying weekly duration of time engaged in each activity by the corresponding MET, and summing the values across all activities. Height and weight were measured using an automated scale (BF-220; Tanita, Tokyo, Japan). Body mass index (BMI) was calculated as the weight (kg) divided by the square of the height (m^2^).

### Statistical analyses

Baseline characteristics were compared across quartiles of depression score by using one-way analysis of variance (continuous variables) or chi-squared test (categorical variables). The means of age and depression score across quartiles of obesity/metabolic factors were tested by one-way analysis of variance. Multivariable logistic regression was used to estimate odds ratios (ORs) and 95% confidence intervals (95% CIs) for having overweight/obesity, high VFA, high SFA, high fasting glucose, high fasting insulin and high HOMA-IR for each quartile of depression score, with the lowest quartile as the reference. Variables adjusted for in model 1 were age (years, continuous), smoking (never, past or current), alcohol use (nondrinker, drinker consuming <1, 1–1.9 or ≥2 *go* of sake-equivalent ethanol per day; 1 *go* of sake contains 23 g ethanol) and leisure time and commuting physical activity (≥400 or <400 METs-min/week). BMI (kg/m^2^, continuous) was additionally adjusted for in model 2. SFA (cm^2^, continuous) was added to model 1 when estimating OR for high VFA (model 3), whereas VFA (cm^2^, continuous) was added to model 1 when estimating OR for high SFA (model 4). We assessed *p* for trend with 1 to 4 being assigned to increasing quartile of depression score and treated as continuous. All analyses were performed using SPSS for Windows, Version 16.0 (SPSS Inc., IL, USA).

## Results

As shown in Table 2, participants with high depression scores were significantly younger and tended not to engage in physical activity than those with low depression scores. Participants in the highest quartile of depression score showed significantly higher SFA, fasting insulin and HOMA-IR levels than those in the lowest quartile of depression score.

**Table 2:** Characteristics of study participants.

	Quartile of depression score			
	Q1	Q2	Q3	Q4
Depression score (mean±SD)	<16 (13.9 ± 0.8)	16–19 (17.4 ± 1.1)	20–23 (21.4 ± 1.1)	≥24 (28.3 ± 4.8)
No. of participants	1,277	1,104	884	1,068
Age (years)	52.2 ± 7.9	50.0 ± 8.0**	48.1 ± 7.8**	46.0 ± 7.4**
Smoking (%)				
Never	29.8	28.2	27.5	27.6
Past	34.8	32.2	31.3	27.6
Current	35.4	39.7	41.2	44.8
Alcohol use (%)				
Nondrinker	18.8	22.3	23.1	24
Drinking <1 *go* per day	43.1	41.5	41.5	42.2
Drinking 1–1.9 *go* per day	28.5	24.0	25.2	20.9
Drinking ≥2 *go* per day	9.6	12.2	10.2	12.9
Physical activity (%)^a^	58.5	52.3	51.7	44.9
Body mass index (kg/m^2^)	24.0 ± 2.8	23.9 ± 2.9	24.1 ± 3.1	24.3 ± 3.2
Visceral fat area (cm^2^)	120 ± 52	121 ± 52	121 ± 52	118 ± 53
Subcutaneous fat area (cm^2^)	129 ± 56	130 ± 56	136 ± 59*	139 ± 65**
Fasting plasma glucose (mg/dL)	103 ± 13	103 ± 13	102 ± 17	102 ± 14
Hemoglobin A1c (%)	5.8 ± 0.4	5.8 ± 0.4	5.8 ± 0.5	5.7 ± 0.5
Fasting insulin (μU/mL)	5.9 ± 4.1	5.9 ± 3.8	6.3 ± 4.1*	6.7 ± 4.4**
HOMA-IR	1.53 ± 1.23	1.52 ± 1.03	1.63 ± 1.21	1.72 ± 1.30**

HOMA-IR: homeostasis model assessment of insulin resistance, Data are means ± standard deviation unless stated otherwise

^a^Combined activities on leisure and commuting (walking) to and from work ≥400 MET-min per week

*P <0.05 and

**P <0.01 (versus Q1)

Significant difference across depression categories was observed for smoking, alcohol use and physical activity by chi-squared test (*P* <0.01).

As shown in Table 3, participants in the highest quartile of BMI or SFA were significantly younger than those in the lowest BMI or SFA. In contrast, participants with the high VFA (Q2 to Q4) were significantly older than those with the lowest VFA. In univariate analysis, participants in the highest quartile of SFA had significantly higher depression score than those in the lowest SFA. Participants with high level of fasting insulin (Q2 to Q4) and HOMA-IR (Q2 to Q4) had significantly higher depression score than those with the lowest.

**Table 3:** Mean value of age and depression score in each quartile of obesity indices/markers of insulin resistance.

	Quartile of obesity indices/markers of insulin resistance			
	Q1	Q2	Q3	Q4
**Body mass index (kg/m**^**2**^**)**	<22.2	22.2–23.9	24–25.7	≥25.8
No. of participants	1,111	1,104	1,035	1,083
Age (years)	49.5 ± 8.5	49.7 ± 8.0	49.9 ± 7.9	48.1 ± 8.0**
Depression score	19.6 ± 5.8	19.9 ± 5.9	19.8 ± 6.0	20.2 ± 6.4
**Visceral fat area (cm**^**2**^**)**	<85	85–119	120–154	≥155
No. of participants	1,093	1,095	1,067	1,078
Age (years)	47.7 ± 8.9	48.8 ± 8.3*	49.9 ± 7.5**	50.8 ± 7.5**
Depression score	20.0 ± 6.2	19.9 ± 6.1	19.8 ± 6.1	19.8 ± 5.8
**Subcutaneous fat area (cm**^**2**^**)**	<97	97–127	128–164	≥165
No. of participants	1,086	1,084	1,081	1,082
Age (years)	50.1 ± 8.4	50.1 ± 8.1	49.9 ± 7.3	47.0 ± 7.7**
Depression score	19.4 ± 5.9	19.5 ± 5.7	19.9 ± 5.8	20.7 ± 6.6**
**Fasting plasma glucose (mg/dL)**	<96	96–100	101–107	≥108
No. of participants	1,185	1,019	1,107	1,022
Age (years)	47.6 ± 8.6	48.7 ± 8.3**	49.4 ± 7.7**	51.5 ± 7.3**
Depression score	20.2 ± 6.1	20.0 ± 6.1	19.7 ± 6.0	19.6 ± 6.0
**Fasting insulin (μU/mL)**	<3.7	3.7–5.2	5.3–7.6	≥7.7
No. of participants	1,134	1,069	1,069	1,061
Age (years)	51.0 ± 8.1	49.6 ± 8.1**	48.7 ± 8.1**	47.7 ± 8.0**
Depression score	19.0 ± 5.4	19.8 ± 6.1**	20.0 ± 6.3**	20.6 ± 6.2**
**HOMA-IR**	<0.87	0.87–1.29	1.30–1.96	≥1.97
No. of participants	1,081	1,086	1,090	1,076
Age (years)	50.9 ± 8.1	49.3 ± 8.2**	48.5 ± 8.1**	48.3 ± 7.8**
Depression score	19.1 ± 5.5	19.8 ± 6.0*	20.2 ± 6.2**	20.5 ± 6.3**

HOMA-IR: homeostasis model assessment of insulin resistance, Data are means ± standard deviation unless stated otherwise

*P <0.05 and

**P <0.01 (versus Q1).

Table 4 shows the associations between obesity indices, markers of insulin resistance and the depression score. Depression score was significantly associated with VFA and fasting insulin in men; multivariable-adjusted ORs (95% CI) of high VFA for the lowest through highest quartile of depression score were 1 (reference), 1.18 (0.97–1.42), 1.25 (1.02–1.54), 1.23 (1.01–1.51), respectively (*P* for trend = 0.03), and the corresponding figures for high fasting insulin were 1 (reference), 0.98 (0.80–1.19), 1.12 (0.91–1.38), and 1.29 (1.06–1.57), respectively (*P* for trend <0.01). There was a suggestion of a positive association between HOMA-IR and depression score (*P* for trend = 0.06). In contrast, SFA was not associated with depression score (*P* for trend >0.2). These associations were virtually unchanged after additional adjustment for BMI. The association between VFA and depression score was somewhat attenuated after additional adjustment for SFA and became statistically not significant. The Pearson correlation coefficient between BMI and VFA was 0.641 (*P* <0.01), between BMI and SFA was 0.778 (*P* <0.01), and between VFA and SFA was 0.537 (*P* <0.01).

**Table 4:** Association of elevated depressive symptoms with obesity indices/markers of insulin resistance.

	Quartile of depression score				*P* for trend
	Q1 (<16)	Q2 (16–19)	Q3 (20–23)	Q4 (≥24)	
Overweight/Obesity					
No. of cases	435	371	298	386	
Age-adjusted OR (95%CI)	1	0.99 (0.83–1.19)	0.95 (0.79–1.13)	0.92 (0.77–1.12)	>0.2
OR^a^ (95%CI)	1	0.95 (0.80–1.12)	0.93 (0.77–1.11)	1.00 (0.84–1.19)	>0.2
High VFA					
No. of cases	308	285	227	258	
Age-adjusted OR (95%CI)	1	1.18 (0.98–1.43)	1.26 (1.03–1.54)	1.25 (1.02–1.52)	0.02
OR^a^ (95%CI)	1	1.18 (0.97–1.42)	1.25 (1.02–1.54)	1.23 (1.01–1.51)	0.03
OR^b^ (95%CI)	1	1.34 (1.08–1.67)	1.39 (1.10–1.76)	1.26 (1.00–1.60)	0.04
OR^c^ (95%CI)	1	1.21 (0.99–1.49)	1.24 (0.99–1.55)	1.16 (0.93–1.44)	0.17
High SFA					
No. of cases	280	257	237	308	
Age-adjusted OR (95%CI)	1	0.98 (0.81–1.20)	1.10 (0.90–1.35)	1.12 (0.92–1.36)	0.18
OR^a^ (95%CI)	1	0.97 (0.80–1.19)	1.09 (0.89–1.34)	1.10 (0.90–1.34)	>0.2
OR^b^ (95%CI)	1	1.16 (0.90–1.51)	1.23 (0.93–1.62)	1.16 (0.89–1.51)	>0.2
OR^d^ (95%CI)	1	0.91 (0.73–1.14)	0.96 (0.76–1.21)	1.01 (0.81–1.26)	>0.2
High fasting glucose					
No. of cases	271	232	153	192	
Age-adjusted OR (95%CI)	1	1.10 (0.90–1.35)	0.96 (0.76–1.20)	1.12 (0.90–1.40)	>0.2
OR^a^ (95%CI)	1	1.11 (0.91–1.41)	0.96 (0.76–1.20)	1.13 (0.91–1.41)	>0.2
OR^b^ (95%CI)	1	1.15 (0.93–1.41)	0.96 (0.76–1.21)	1.12 (0.89–1.40)	>0.2
High fasting insulin					
No. of cases	275	245	227	314	
Age-adjusted OR (95%CI)	1	0.98 (0.80–1.19)	1.13 (0.92–1.38)	1.28 (1.06–1.56)	<0.01
OR^a^ (95%CI)	1	0.98 (0.80–1.19)	1.12 (0.91–1.38)	1.29 (1.06–1.57)	<0.01
OR^b^ (95%CI)	1	1.06 (0.85–1.32)	1.16 (0.92–1.47)	1.33 (1.07–1.67)	<0.01
High HOMA-IR					
No. of cases	294	263	213	306	
Age-adjusted OR (95%CI)	1	1.01 (0.83–1.22)	1.00 (0.82–1.21)	1.27 (1.03–1.55)	0.05
OR^a^ (95%CI)	1	1.00 (0.83–1.23)	0.99 (0.81–1.21)	1.26 (1.02–1.54)	0.06
OR^b^ (95%CI)	1	1.09 (0.88–1.35)	0.99 (0.79–1.23)	1.28 (1.02–1.62)	0.11

VFA: visceral fat area, SFA: subcutaneous fat area, HOMA-IR: homeostasis model assessment of insulin resistance

OR^a^: (model 1) Adjusted for age, smoking, alcohol drinking, and physical activity

OR^b^: (model 2) Adjusted for age, smoking, alcohol drinking, physical activity and body mass index

OR^c^: (model 3) Adjusted for age, smoking, alcohol drinking, physical activity and subcutaneous fat area

OR^d^: (model 4) Adjusted for age, smoking, alcohol drinking, physical activity and visceral fat area

Definition (cutoff) of ‘High’ group (outcome): overweight/obesity ≥25 kg/m^2^, fasting plasma glucose ≥110 mg/dL

highest one-fourth for VFA (≥155 cm^2^), SFA (≥165cm^2^), fasting insulin (≥7.7 μU/mL) and HOMA-IR (≥1.97).

## Discussion

In the present analysis of male employees without treated diabetes and mental disorder, VFA and fasting insulin, but not SFA, were significantly associated with EDS after multivariable adjustment. This study is among a few large-scale population-based studies that reported depressive symptoms in relation to type of abdominal fat deposition and, to our knowledge, the first such study in Asia.

Smaller scale studies (n <500) that assessed abdominal fat accumulation using a CT or magnetic resonance tomography reported a significant association between depression and visceral fat deposition in women [17–19]. In a large CT study among a middle-aged population (n = 3,299), Murabito et al found a significantly increased odds of depressive symptoms in women, but not in men, with high visceral fat accumulation [20]. Vogelzangs et al showed that high visceral fat at baseline was associated with a significantly increased risk of depression onset in older men, but not in older women [21]. Remigio-Baker et al. reported a significant relationship between EDS and visceral adiposity in men, but not in women [22]. Although sex-difference in association has been documented, the present result together with previous data suggest a role of VFA in mood disorder.

In a large study of middle-aged men and women [20], subcutaneous adipose tissue was not significantly associated with EDS in both sexes. Similarly, the present study did not find a significant association between SFA and EDS in men after adjustment for age, denying a role of subcutaneous fat deposition in the etiology of depression. The result is compatible with a weaker association of SFA than VFA with markers of inflammation and insulin resistance [29,30], which are thought to be potential mechanisms underlying the relationship between depression and obesity.

The association between depression and insulin resistance has been inconsistent, with some studies showing a positive association [31,32] while others reporting no association [33,34]. In the present study, odds of high fasting insulin and high HOMA-IR increased by nearly 30% in the highest group of depression score in participants. The finding among participants agrees with those of a meta-analysis [24], reporting a small but significant overall association between depression and insulin resistance.

Several mechanisms whereby depression induces obesity and impaired glucose metabolism have been suggested. Depression is associated with disruption of HPA-axis, which increases blood concentrations of cortisol, leading to insulin resistance [9]. Cortisol and insulin stimulate lipid uptake by activating lipoprotein lipase, and this process is facilitated by high concentrations of cortisol-activate enzyme 11-β-hydroxysteroid dehydrogenase type 1 and glucocorticoid receptors. Visceral fat contains a higher concentration of glucocorticoid receptors and has greater lipoprotein lipase activity than subcutaneous fat [35,36]. In addition, chronic stress causes glucocorticoid receptor resistance that results in a failure to down-regulate response to inflammation [37], which plays an important role in the development of both insulin resistance [38] and obesity [39]. Pro-inflammatory cytokines (such as tumor necrosis factor-α and interleukin-6) have been reported to be increased in depression [18,40] and have also been implicated in the development of insulin resistance [38]. Depression could deteriorate lifestyle behaviors including physical activity, dietary habit and sleep rhythm [12]. Similar mechanisms in the opposite direction have also been suggested. Obesity may induce inflammation [41] and HPA-axis dysregulation [42]. Obesity increases risk of insulin resistance and diabetes, which induces cerebral microvascular damage and increase the risk of depression [43,44].

The present study has some limitations. First, the depression score was not assessed based on a validated questionnaire. However, questions used in the calculation of the present depression score were similar to those of standard questionnaires and the present score is well correlated with SDS score. Second, this study is cross-sectional and thus cannot provide data for causal inference. Third, we did not adjust for potentially important variables including diet, which may confound the association. Finally, the study participants were employees of a large-scale company. Caution should be exercised in the application of the present results to a population with a different background.

## Conclusion

EDS was associated with VFA and markers of insulin resistance in middle-aged Japanese men. Longitudinal studies are required to confirm the present cross-sectional finding and to establish temporality of these relationships.
